# An integrated pan‐cancer analysis of TFAP4 aberrations and the potential clinical implications for cancer immunity

**DOI:** 10.1111/jcmm.16147

**Published:** 2020-12-29

**Authors:** Jian‐Nan Liu, Xiang‐Shuo Kong, Ping Sun, Rui Wang, Wang Li, Qi‐Feng Chen

**Affiliations:** ^1^ Department of Oncology Yantai Yuhuangding Hospital Yantai China; ^2^ Department of Respiratory Oncology Fushan district people's hospital Yantai China; ^3^ Department of Medical Imaging and Interventional Radiology Sun Yat‐sen University Cancer Center Guangzhou China; ^4^ State Key Laboratory of Oncology in South China Guangzhou China; ^5^ Collaborative Innovation Center for Cancer Medicine Guangzhou China

**Keywords:** immune infiltration, pan‐cancer, prognostic biomarker, TFAP4

## Abstract

Studies have shown that transcription factor activating enhancer binding protein 4 (TFAP4) plays a vital role in multiple types of cancer; however, the TFAP4 expression profile is still unknown, as is its value within the human pan‐cancer analysis. The present study comprehensively analysed TFAP4 expression patterns from 33 types of malignancies, along with the significance of TFAP4 for prognosis prediction and cancer immunity. TFAP4 displayed inconsistent levels of gene expression across the diverse cancer cell lines, and displayed abnormal expression within most malignant tumours, which closely corresponded to overall survival. More importantly, the TFAP4 level was also significantly related to the degree of tumour infiltration. TFAP4 was correlated using gene markers in tumour‐infiltrating immune cells and immune scores. TFAP4 expression was correlated with tumour mutation burden and microsatellite instability in different cancer types, and enrichment analyses identified TFAP4‐associated terms and pathways. The present study comprehensively analysed the expression of TFAP4 across 33 distinct types of cancers, which revealed that TFAP4 may possibly play a vital role during cancer formation and development. TFAP4 is related to differing degrees of immune infiltration within cancers, which suggests the potential of TFAP4 as an immunotherapy target in cancers. Our study demonstrated that TFAP4 plays an important role in tumorigenesis as a prognostic biomarker, which highlights the possibility of developing new targeted treatments.

## 1 INTRODUCTION

Over the past few decades, the morbidity of malignant tumours has increased at an alarming rate, which may be attributed to increased life expectancy, altered lifestyle habits, and interactions between genetic factors and external agents (physical, chemical and biological carcinogens). Malignant tumours are one of the leading causes of death worldwide, with low therapeutic success in both developed and developing countries.^1^ Pan‐cancer analysis has been widely utilized in cancer research to shed more light on the common features, heterogeneities, emerging themes and analytical breadth of various human malignancies.^2^ Pan‐cancer analysis is the analysis of the molecular abnormalities of various types of cancer, which can identify any common features and heterogeneities during vital biological processes that are under dysregulation as the result of diverse cancer cell lineages. Pan‐cancer analysis projects, such as the Cancer Cell Line Encyclopedia (CCLE) and The Cancer Genome Atlas (TCGA), have been created based on the assessment of different human cancer cell lines and tissues at epigenomic, genomic, proteomic and transcriptomic levels.^3^, ^4^, ^5^ Recently, pan‐cancer analysis has been used to identify certain functional and pathway genes, which allows for a comprehensive and thorough understanding of human cancers. For example, tumour hypoxia‐associated multiomic molecular characteristics have been investigated, and it has been suggested that some molecular alterations can be correlated with drug sensitivity or resistance to antitumour agents. This helps to comprehensively understand tumour hypoxia at the molecular level and has certain implications for cancer treatment in clinical practice.^6^ New data on FOXM1 up‐regulation frequency, aetiology and outcomes in human cancers have been defined from 33 TCGA‐derived cancers.^7^ The information obtained from these cancers has revealed lncRNA‐mediated dysregulation within the cancer at a system level, and provides a valuable approach and resources to investigate lncRNA functions in the context of cancer.^8^ Characterizing immune phenotype occurrence frequency and variability in a variety of types of cancer helps to understand the immune status of untreated cancers, and this approach has been used in more than 9000 TCGA‐derived cancer gene expression data sets.^9^ Therefore, pan‐cancer analysis can illustrate patterns beneficial for developing combination and individualized therapies for the treatment of various cancers.

Transcription factor activating enhancer binding protein 4 (TFAP4) is involved in cancer proliferation, metastasis, differentiation, angiogenesis and other biological functions.^10^ In recent years, it has been suggested that the overexpression of TFAP4 may indicate a poor prognosis for various cancers, including hepatocellular carcinoma (HCC), non‐small cell lung carcinoma (NSCLC), prostate cancer (PCa), colorectal cancer (CRC) and gastric cancer (GC).^11^, ^12^, ^13^, ^14^, ^15^ According to our prior research, TFAP4 plays a role as an efficient prognostic biomarker, which also activates the PI3K/AKT signal transduction pathway to enhance the metastasis and invasion of HCC.^16^ Other studies have been carried out to examine the proliferation, overexpression or mutation of TFAP4 in specific types of cancer, but those studies had low sample sizes and diverse methods. Additionally, research on TFAP4 has mainly focused on an individual or limited number of types of cancers, and no available studies have comprehensively examined several types of cancers simultaneously to identify their similarities and differences. This information is of great importance for understanding the roles of TFAP4 in various cancers, so a comprehensive analysis is urgently needed.

To that end, and taking advantage of the large data sets from TCGA, the present study aimed to examine TFAP4 expression profiles and their prognostic significance among human cancers. Additionally, the associations between TFAP4 and the levels of tumour infiltration, tumour mutational burden (TMB) and microsatellite instability (MSI) were analysed for different types of tumour using correlation analysis. Gene set enrichment analysis (GSEA) was conducted to investigate any possible underlying mechanisms. The results of the present study can help to understand vital parts of TFAP4 in the context of tumours, reveal the possible association of TFAP4 with tumour‐immune interactions and illustrate the potential mechanism.

## 2 MATERIALS AND METHODS

### 2.1 Patient data sets and processing

TCGA, a cornerstone of the cancer genomics projects, had characterized more than 20,000 primary cancer samples and corresponding non‐carcinoma samples from 33 types of cancers. In the present study, the TCGA‐processed level 3 RNA‐sequencing data sets, along with the corresponding clinical annotations, were obtained using the University of California Santa Cruz (UCSC) cancer genome browser (https://tcga.xenahubs.net, accessed April 2020). The CCLE public project has comprehensively characterized a tremendous number of human tumour models both genetically and pharmacologically (https://portals.broadinstitute.org/ccle). To examine differential gene expression in cancers on a larger scale, the CCLE database, which contains RNA‐sequencing data sets for over 1,000 cell lines, was used. For this research, only open‐access data were used, which precluded the requirement of approval of the Ethics Committee.

### 2.2 Screening of TFAP4 differential expression and its survival‐associated cancers

To compare gene expression levels between the cancerous and adjacent normal samples, data regarding TFAP4 gene expression were extracted from the 33 TCGA cancer types to form an expression matrix, as shown in Table S1. Thereafter, the expression matrix and clinical information were matched by patient ID. Afterwards, a univariate Cox model was used to calculate any association between gene expression levels and patient survival, where a difference of *P* < .05 for TFAP4 in a specific cancer was deemed statistically significant. The survival‐associated forest plot was further drawn, and a Kaplan‐Meier (KM) analysis was conducted to compare the overall survival (OS) for TCGA cancer patients stratified according to the median TFAP4 expression level, using the log‐rank test.

### 2.3 TFAP4 and tumour immunity

The Tumour Immune Estimation Resource (TIMER, https://cistrome.shinyapps.io/timer/) represents the integrated approach to systemically analysing the immune infiltrates of different types of cancers.^17^ In TIMER, the deconvolution statistical approach is used for inferring tumour‐infiltrating immunocyte levels based on gene expression data.^18^ Using the TIMER algorithm, we examined the associations between TFAP4 levels and six different immune infiltrate levels (CD4 + T cells, CD8 + T cells, B cells, neutrophils, dendritic cells and macrophages).

The relative subsets of RNA transcripts (CIBERSORT) were used to calculate relative fractions of 22 types of leucocyte. CIBERSORT is a highly accurate metagene tool, which precisely estimates 22 phenotypes of human immunocytes, as previously reported for all TCGA samples ^19^ (Table S2). For the present study, the association of TFAP4 expression with each leucocyte phenotype across 33 cancer types was computed.

Additionally, we examined the associations of TFAP4 levels with tumour‐infiltrating immunocyte gene markers selected based on previous research.^20^, ^21^, ^22^ The correlation analysis generated the estimated statistical significance and Spearman's correlation coefficient. Then, an expression heat map was plotted for gene pair within the specific type of cancer.

The estimation of stromal and immune cells in malignant tumour tissues using expression data (refer to ESTIMATE for short) represents an approach that uses gene expression profiles to predict the purity of both tumours and the infiltrating stromal cells/immunocytes within tumour tissues.^23^ The ESTIMATE algorithm produces three scores on the basis of single sample Gene Set Enrichment Analysis (ssGSEA), including 1) stromal score, which determines stromal cells within the tumour tissues, 2) immune score, which assesses immunocyte infiltration within the tumour tissues, and 3) estimate score, which can infer the purity of tumour. In this study, we used the ESTIMATE algorithm to estimate both immune and stromal scores (Table S3) for tumour tissues according to the corresponding transcription data. Then, we calculated the correlations between these scores and TFAP4 expression.

TMB measures the mutation number in a specific cancer genome. Numerous studies have explored the significance of using TMB as a biomarker for predicting which patients would be most responsive to checkpoint inhibitors.^24^ We downloaded the somatic mutation data for all TCGA patients (https://tcga.xenahubs.net), calculated their TMB scores (Table S4) and then determined the correlation between TMB and TFAP4. MSI is characterized by the widespread length polymorphisms of microsatellite sequences due to DNA polymerase slippage. Recently, it has been suggested that patients with high‐MSI cancers gain benefits from immunotherapy, and MSI has been utilized as an indicator of genetic instability for the cancer detection index.^25^ We computed the MSI score for each patient, as shown in Table S5, and subsequently performed a correlation analysis between MSI and TFAP4.

### 2.4 Gene set enrichment analysis

Using JAVA (http://software.broadinstitute.org/gsea/index.jsp), we conducted GSEA to assess for possible underlying mechanisms based on the ‘Molecular Signatures Database’ of c5.all.v7.1.symbols and c2.cp.kegg.v7.1.symbols. With a random sample permutation number of 100 and the threshold of significance as *P* < .05, Bioconductor (http://bioconductor.org/) and R software (http: //r‐project. org/) were used to plot enrichment maps to visualize our results.

### 2.5 Statistical methods

For the present study, we selected clinical indicators, including OS, disease‐specific survival (DSS), progression‐free interval (PFI) and disease‐free interval (DFI). OS was defined as the duration from the date of diagnosis to death, from any cause. Unlike OS, for DSS, patients who died from causes other than the specified disease were not counted. PFI was defined as disease progression or death, again, from any cause. Unlike PFI, patients who died from causes other than the specified disease were not counted in the DFI.

The Wilcoxon log‐rank test was used to determine the presence or absence of a markedly increased sum of gene expression z‐scores for cancerous tissues, as compared to adjacent normal tissues. The difference in TFAP4 expression between different tumour stages was compared using the Kruskal‐Wallis test. Survival was analysed using the KM curves, log‐rank test and Cox proportional hazards regression model. Spearman's test was used for correlation analysis. R language (version 3.6.0; R Foundation) was used for all analyses. A two‐sided *P*‐value < .05 indicated a statistically significant difference.

## 3 RESULTS

### 3.1 Pan‐cancer expression landscape of TFAP4

**Figure 1:**
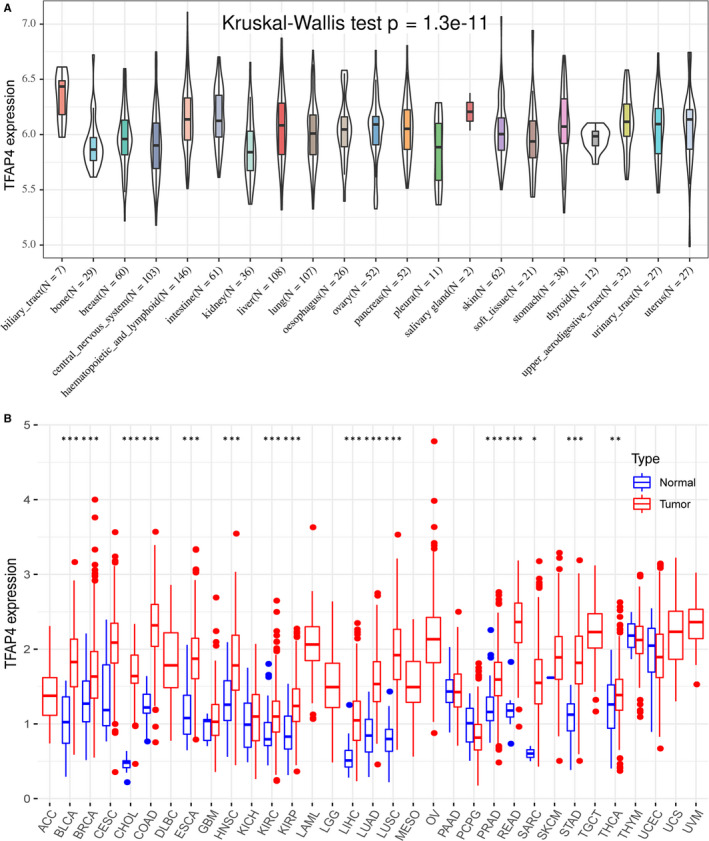
The TFAP4 expression level in human pan‐cancer analyses. (A) The mRNA level of TFAP4 in CCLE. (B) The mRNA level of TFAP4 in TCGA. The blue and red bar graphs indicate normal and tumour tissues, respectively. *P < .05; **P < .01; ***P < .001

According to CCLE analysis results, TFAP4 displayed inconsistent gene expression levels among various cancer cell lines (*P* = 1.3e‐11, Figure 1A), with biliary tract cells showing a relatively higher gene expression. Consistent with kidney cells, which showed a relatively lower gene expression in CCLE, TFAP4 also displayed relatively lower expression in TCGA‐KICH, TCGA‐KIRC and TCGA‐KIRP. For most of the 33 TCGA‐derived cancer types, we detected significantly up‐regulated TFAP4 expression between cancer samples and paired normal samples. Figure 1B shows the TFAP4 expression profiles of the TCGA‐derived samples.

**Figure 2:**
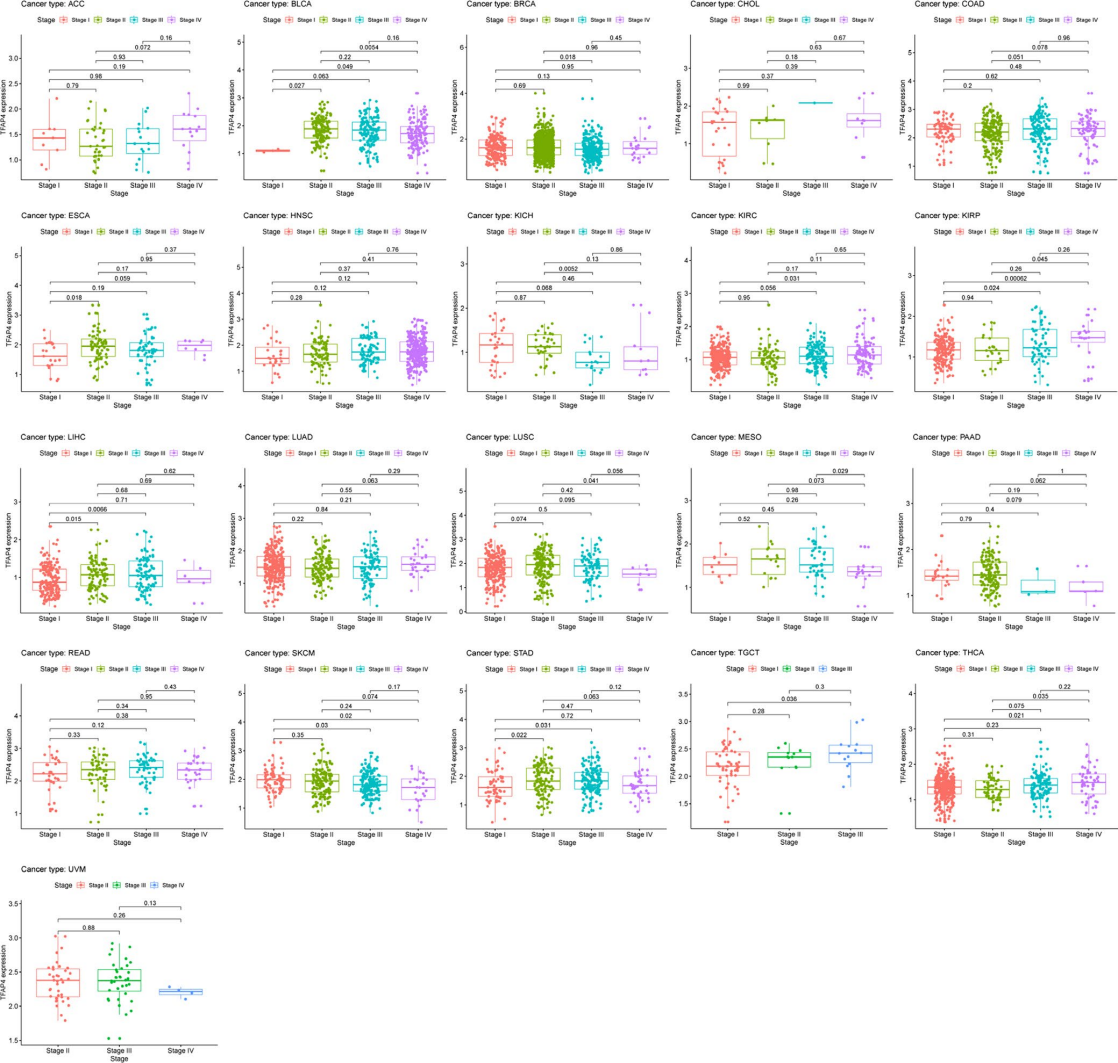
The box plot shows the association of TFAP4 expression with pathological stages for 21 types of cancers

To assess the levels of gene expression for all tumour stages, we compared TFAP4 expression in patients with stage I, II, III or IV tumours. Generally, TFAP4 expression was up‐regulated in advanced tumours in ESCA, KIRC, KIRP,LIHC, LUAD, TGCT and THCA, while it was down‐regulated in advanced tumours in BLCA, BRCA, KICH, LUSC,MESO and SKCM, and was stable in advanced tumours in ACC, CHOL, COAD, HNSC, PAAD, READ, STAD and UVM (Figure 2).

### 3.2 Screening of TFAP4 survival‐associated cancers

**Figure 3:**
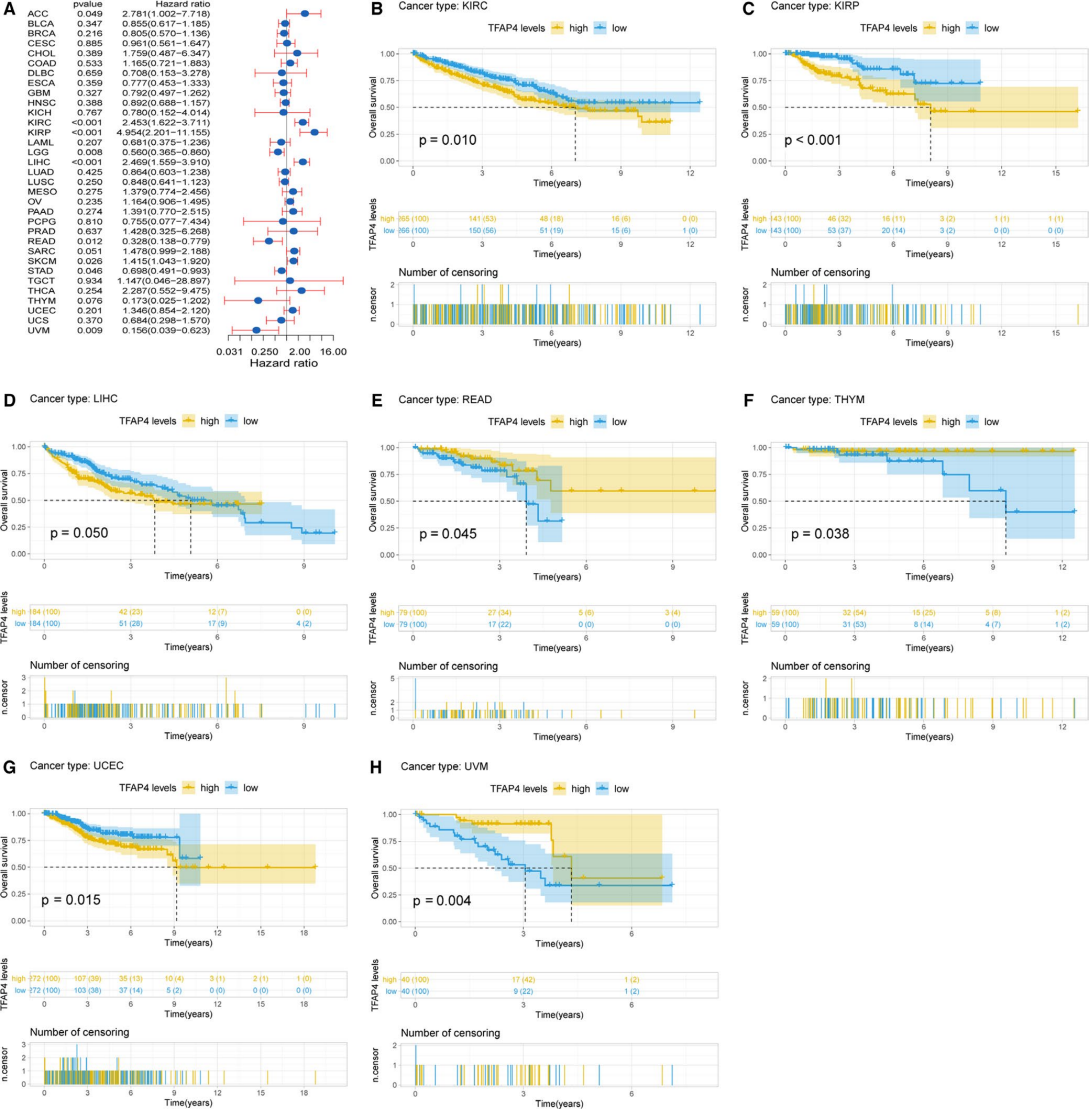
Association of TFAP4 expression with patient overall survival (OS). (A) Forest plot shows the relationship of TFAP4 expression with patient OS. (B‐H) Kaplan‐Meier analyses show the association between TFAP4 expression and OS

In the OS analysis, Cox regression identified that high TFAP4 expression was a risk factor for ACC (*P* = .049), KIRC (*P* < .001), KIRP (*P* < .001), SKCM (*P* = .026) and LIHC (*P* < .001); however, it appeared to be a protective factor in UVM (*P* = .009), READ (*P* = .012), STAD (*P* = .046) and LGG (*P* = .008), as shown in Figure 3A and Table 1. KM analysis showed that patients with higher TFAP4 levels had a shorter OS compared with patients with lower TFAP4 levels in KIRC (*P* = .010), KIRP (*P* < .001), LIHC (*P* = .050) and UCEC (*P* = .015), whereas those with increased TFAP4 levels showed a superior OS to those with decreased TFAP4 levels in READ (*P* = .045), THYM (*P* = .038) and UVM (*P* = .004), as seen in Figure 3B‐H.

**Table 1:** Univariate Cox regression analysis of the association of the TFAP4 level with patients’ survival

Cancer	OS			DSS			DFI			PFI		
	HR	HR(95% CI)	*P*‐value	HR	HR(95% CI)	*P*‐value	HR	HR(95% CI)	*P*‐value	HR	HR(95% CI)	*P*‐value
ACC	2.781	1.002–7.718	.049	2.384	0.81–7.019	.115	10.115	1.863–54.935	.007	2.970	1.297–6.800	.010
BLCA	0.855	0.617–1.185	.347	0.863	0.597–1.248	.433	0.969	0.464–2.027	.934	1.001	0.736–1.363	.993
BRCA	0.805	0.570–1.136	.216	0.688	0.450–1.052	.084	1.314	0.867–1.992	.197	0.881	0.641–1.210	.433
CESC	0.961	0.561–1.647	.885	1.057	0.580–1.925	.856	1.228	0.515–2.923	.643	1.222	0.721–2.072	.456
CHOL	1.759	0.487–6.347	.389	0.999	0.452–2.211	.999	1.053	0.398–2.784	.918	0.888	0.426–1.853	.752
COAD	1.165	0.721–1.883	.533	0.928	0.582–1.478	.752	1.536	0.663–3.559	.317	0.953	0.680–1.335	.780
DLBC	0.708	0.153–3.278	.659	0.957	0.119–7.703	.967	0.846	0.019–38.286	.932	2.029	0.545–7.556	.291
ESCA	0.777	0.453–1.333	.359	0.743	0.391–1.410	.363	0.944	0.421–2.116	.888	1.073	0.687–1.676	.756
GBM	0.792	0.497–1.262	.327	0.822	0.479–1.411	.477	/	/	/	0.494	0.303–0.803	.004
HNSC	0.892	0.688–1.157	.388	0.771	0.568–1.048	.096	0.679	0.323–1.427	.307	0.862	0.668–1.112	.252
KICH	0.780	0.152–4.014	.767	0.417	0.079–2.211	.304	0.188	0.014–2.430	.200	0.394	0.102–1.528	.178
KIRC	2.453	1.622–3.711	<.001	2.935	1.839–4.686	<.001	0.834	0.248–2.803	.769	2.295	1.556–3.386	<.001
KIRP	4.954	2.201–11.155	<.001	4.706	1.943–11.397	.001	1.816	0.573–5.758	.311	2.083	1.066–4.068	.032
LAML	0.681	0.375–1.236	.207	/	/	/	/	/	/	/	/	/
LGG	0.560	0.365–0.860	.008	0.541	0.338–0.864	.010	1.235	0.398–3.831	.715	0.553	0.389–0.785	.001
LIHC	2.469	1.559–3.910	<.001	1.893	1.110–3.228	.019	1.872	1.276–2.747	.001	1.941	1.376–2.740	<.001
LUAD	0.864	0.603–1.238	.425	0.814	0.559–1.186	.284	1.360	0.885–2.090	.161	0.963	0.724–1.280	.793
LUSC	0.848	0.641–1.123	.250	0.615	0.432–0.874	.007	0.872	0.566–1.344	.536	0.721	0.551–0.944	.017
MESO	1.379	0.774–2.456	.275	1.778	0.791–4.000	.164	0.935	0.108–8.081	.951	1.500	0.795–2.832	.211
OV	1.164	0.906–1.495	.235	1.167	0.893–1.525	.259	1.008	0.730–1.393	.960	0.956	0.749–1.220	.715
PAAD	1.391	0.770–2.515	.274	1.109	0.568–2.163	.762	0.900	0.233–3.472	.878	1.074	0.624–1.846	.797
PCPG	0.755	0.077–7.434	.810	1.421	0.112–18.038	.786	0.165	0.005–4.975	.300	0.719	0.183–2.826	.636
PRAD	1.428	0.325–6.268	.637	4.347	0.515–36.663	.177	4.107	1.728–9.761	.001	2.273	1.383–3.738	.001
READ	0.328	0.138–0.779	.012	0.444	0.175–1.126	.087	0.868	0.196–3.844	.852	0.795	0.418–1.510	.483
SARC	1.478	0.999–2.188	.051	1.525	0.999–2.329	.051	1.140	0.727–1.787	.568	1.131	0.813–1.573	.466
SKCM	1.415	1.043–1.920	.026	1.484	1.068–2.060	.019	/	/	/	1.093	0.841–1.419	.506
STAD	0.698	0.491–0.993	.046	0.752	0.489–1.156	.193	0.714	0.381–1.337	.293	0.820	0.574–1.171	.275
TGCT	1.147	0.046–28.897	.934	2.056	0.074–57.100	.671	1.670	0.586–4.760	.337	1.037	0.406–2.651	.939
THCA	2.287	0.552–9.475	.254	8.027	1.536–41.948	.014	1.031	0.351–3.027	.955	2.789	1.393–5.583	.004
THYM	0.173	0.025–1.202	.076	5.807	0.129–261.867	.365	/	/	/	0.671	0.180–2.500	.552
UCEC	1.346	0.854–2.120	.201	1.608	0.927–2.790	.091	2.056	1.166–3.626	.013	1.681	1.146–2.466	.008
UCS	0.684	0.298–1.570	.370	0.879	0.378–2.043	.764	1.148	0.253–5.217	.858	0.924	0.433–1.971	.837
UVM	0.156	0.039–0.623	.009	0.119	0.027–0.513	.004	/	/	/	0.299	0.085–1.055	.061

Abbreviations: DSS, disease‐specific survival; DFI, disease‐free interval; OS, overall survival; PFI, progression‐free interval.

**Figure 4:**
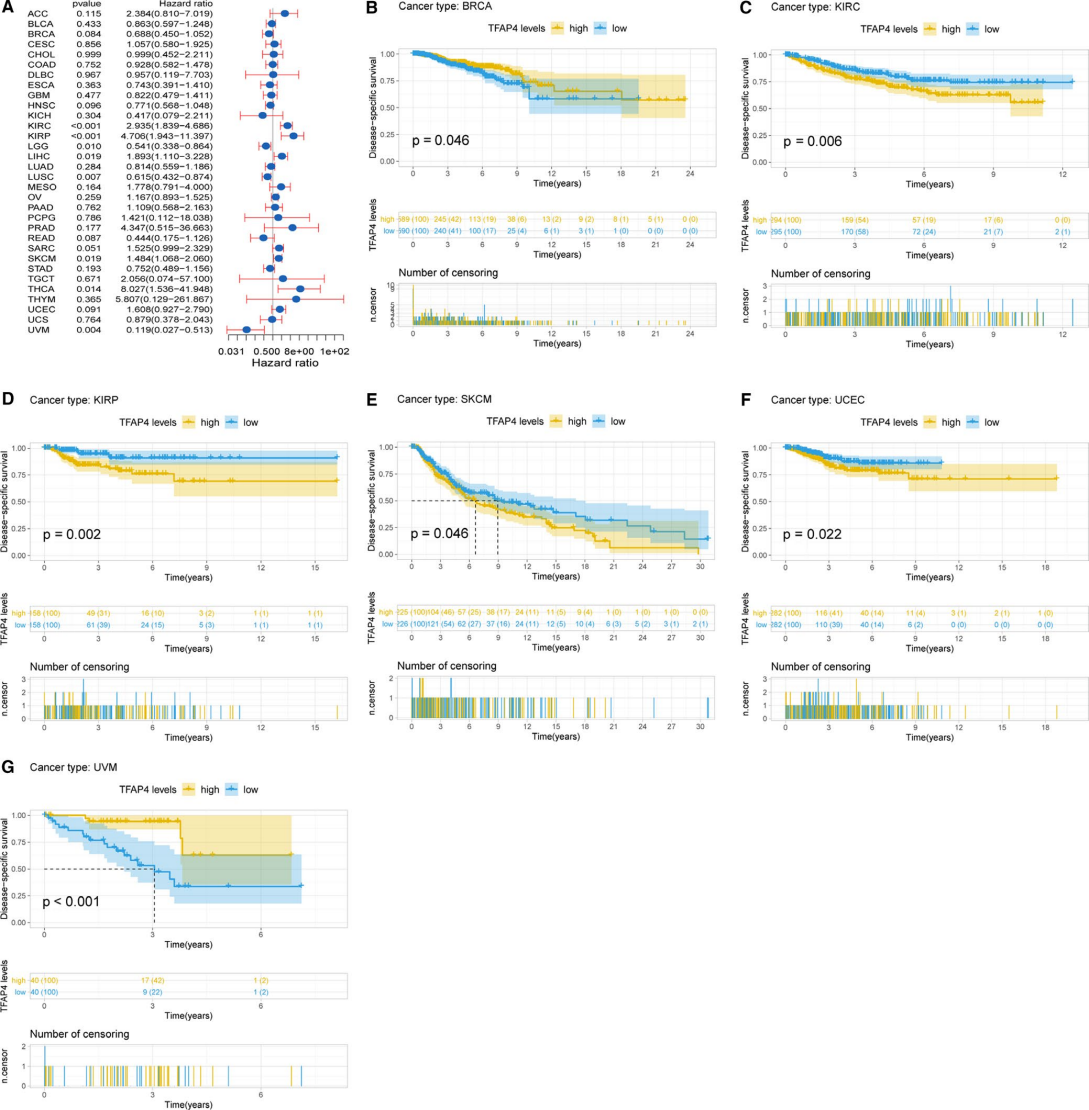
Association of TFAP4 expression with patient disease‐specific survival (DSS). (A) The forest plot shows the relationship of TFAP4 expression with DSS. (B‐G) Kaplan‐Meier analyses show the association between TFAP4 expression and DSS

Cox regression analysis of DSS identified that high TFAP4 expression was a risk factor in KIRC (*P* < .001), LIHC (*P* = .019), KIRP (*P* < .001), SKCM (*P* = .019) and THCA (*P* = .014). However, it was a protective factor in LGG (*P* = .010), LUSC (*P* = .007) and UVM (*P* = .004), as seen in Figure 4A. KM analysis showed that patients with higher TFAP4 expression had poorer DSS than those with lower TFAP4 expression in KIRC (*P* = .006), KIRP (*P* = .002), SKCM (*P* = .046) and UCEC (*P* = .022). Patients with increased TFAP4 levels showed superior DSS to those with decreased TFAP4 levels in BRCA (*P* = .046) and UVM (*P* < .001), as seen in Figure 4B‐G.

**Figure 5:**
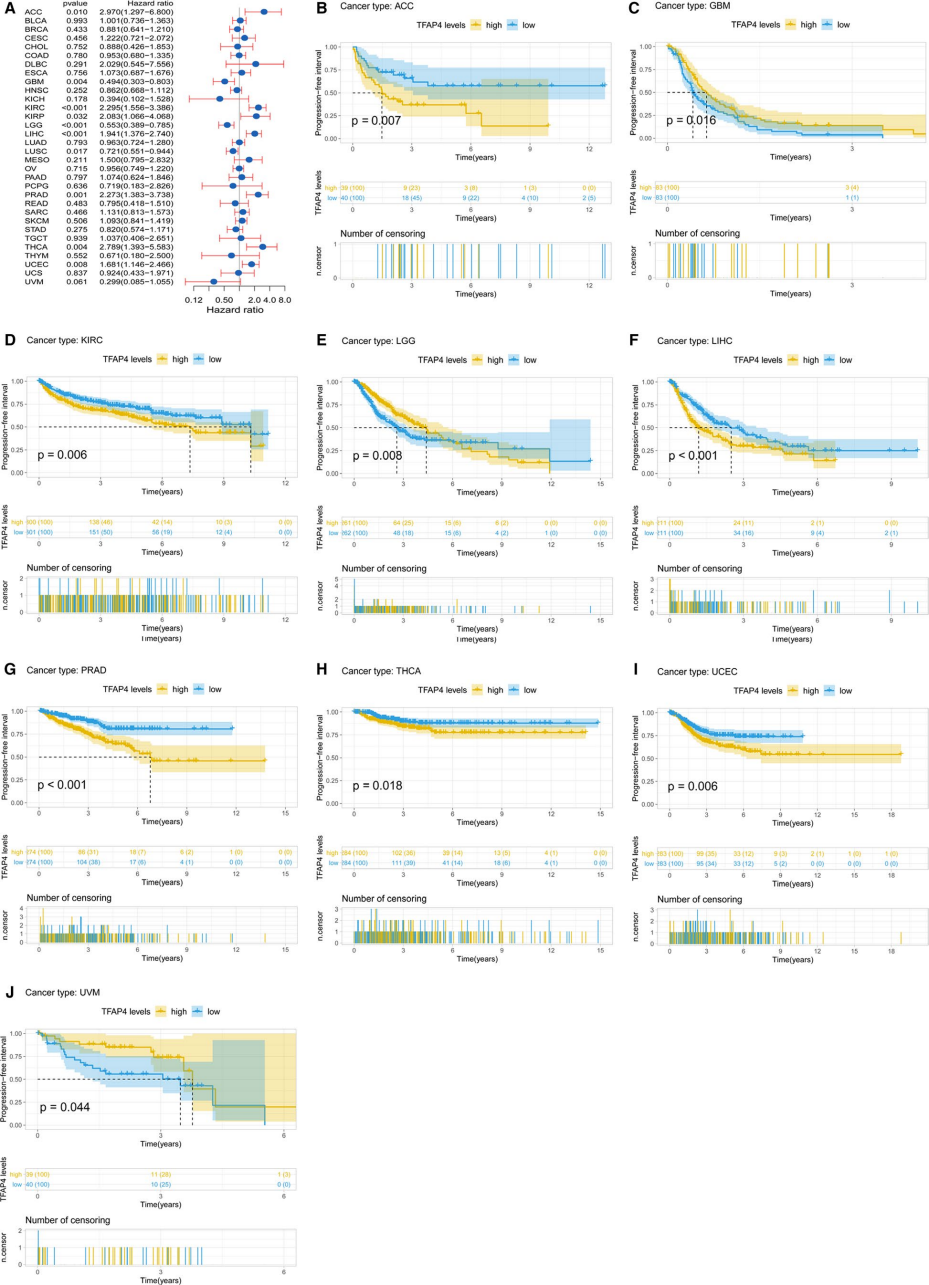
Association of TFAP4 expression with patient progression‐free interval (PFI). (A) The forest plot shows the relationship of TFAP4 expression with PFI. (B‐J) Kaplan‐Meier analyses show the association between TFAP4 expression and PFI

Cox regression analysis of PFI identified high TFAP4 expression as a risk factor in ACC (*P* = .010), KIRP (*P* = .032), KIRC (*P* < .001), PRAD (*P* = .001), LIHC (*P* < .001), THCA (*P* = .004) and UCEC (*P* = .008), while it was a protective factor in GBM (*P* = .004), LGG (*P* < .001) and LUSC (*P* = .017) (Figure 5A). Results of KM analysis showed that patients with higher TFAP4 expression had a poorer OS relative to patients with lower TFAP4 levels in ACC (*P* = .007), KIRC (*P* = .006), LIHC (*P* < .001), PRAD (*P* < .001), THCA (*P* = .018) and UCEC (*P* = .006), whereas patients with increased TFAP4 levels showed a superior OS to those with decreased TFAP4 levels in GBM (*P* = .016), LGG (*P* = .008) and UVM (*P* = .044), as shown in Figure 5B‐J.

**Figure 6:**
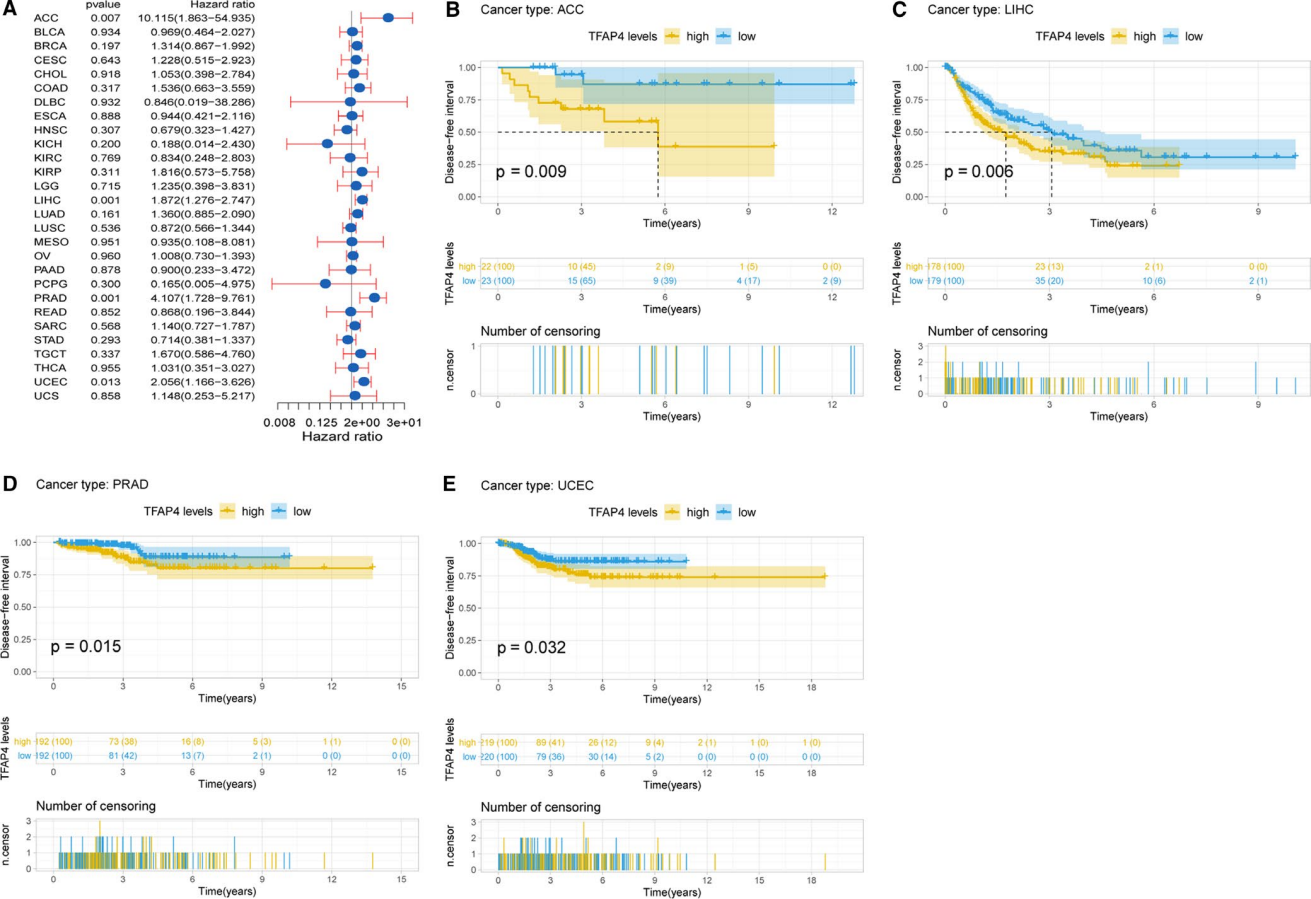
Association of TFAP4 expression with patient disease‐free interval (DFI). (A) The forest plot shows the relationship of TFAP4 expression with DFI. (B‐E) Kaplan‐Meier analyses show the association between TFAP4 expression and DFI

Cox regression analysis of DFI identified that higher TFAP4 expression was a risk factor for ACC (*P* = .007), LIHC (*P* = .001), PRAD (*P* = .001) and UCEC (*P* = .013), as seen in Figure 6A. Of note, KM analysis also showed that higher TFAP4 expression predicted a worse prognosis in these 4 types of cancers (*P* = .009, 0.006, 0.015 and 0.032, respectively) (Figure 6B‐E).

### 3.3 TFAP4 level was related to the level of immune infiltration

**Figure 7:**
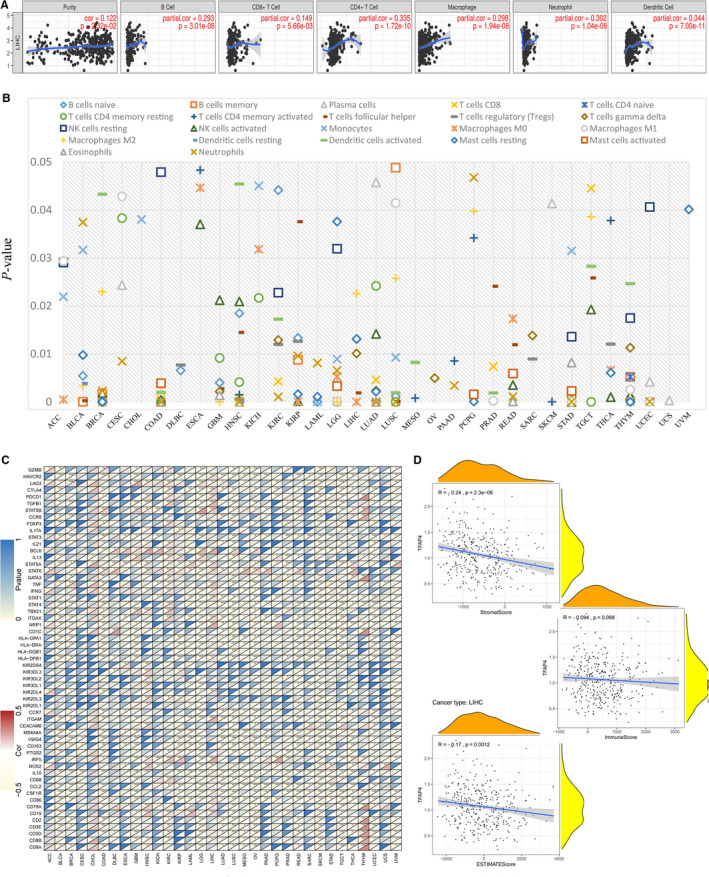
TFAP4 expression is correlated with cancer immunity. (A) TIMER predicts that the TFAP4 level is related to the degree of immune infiltration within HCC. (B) CIBERSORT predicts that TFAP4 expression is correlated with immunocytes. (C) Heat map represents the colour‐coded correlations of immune markers and TFAP4 across 33 types of cancer. For each pair, the left top triangle is coloured to represent the *P‐*value; the right bottom one is coloured to indicate the Spearman correlation coefficient. *P < .05; **P < .01; ***P < .001. (D) The association of TFAP4 with the ESTIMATE scores within HCC

Tumour‐infiltrating lymphocytes (TILs) can serve as independent predictors of sentinel lymph node status and cancer survival. As a result, the present study examined the correlation between TFAP4 levels and the levels of immune infiltration across various types of cancer derived from TIMER. It was found that the TFAP4 level was significantly related to the tumour purity for 25 types of cancer types. Moreover, TFAP4 levels were significantly correlated with CD4 + T and CD8 + T cell infiltration in 15 types of cancer, B cells in 12 types, neutrophils in 16 types, macrophages in 18 types and dendritic cells in 14 types. As TFAP4 was found to show prognostic value in LIHC, the association of TFAP4 level with the degree of immune infiltration in LIHC is shown in Figure 7A, and pan‐cancer associations of TFAP4 levels with the levels of immune infiltration are presented in Figure S1 and Table S6.

Using CIBERSORT, detailed immunocyte compositions of all TCGA patients were calculated, after which the correlations between 22 immunocytes and TFAP4 expression were determined for 33 types of cancer, as seen in Table S7. We found that many immunocytes were significantly correlated with TFAP4 levels. As seen in Figure 7B, in CHOL, OV, UCS and UVM, only one type of immunocyte was correlated with TFAP4 level, while at least two immunocytes were correlated with TFAP4 levels in other cancers.

### 3.4 Correlations of TFAP4 level with immune markers

To investigate the association of TFAP4 expression with different immune infiltrating cells, the relationships between TFAP4 expression and immune markers in a variety of immunocyte types were analysed, as shown in Figure 7C. TFAP4 expression was shown to have a significant correlation with the majority of immune markers in a variety of immunocytes and distinct T cells, as shown in Table S8. TFAP4 was found to be correlated with TIL gene markers in HCC, including those for B cells (CD19 and CD79A), CD8 + T cells (CD8B), monocytes (CD86 and CSF1R), M1 macrophages (NOS2 and IRF5), tumour‐associated macrophages (CD68 and IL10), neutrophils (ITGAM), natural killer cells (KIR2DL4), dendritic cells (HLA‐DPB1, HLA‐DRA,NRP1 and ITGAX), T‐helper 1 cells (STAT1, IFNG and TNF), T‐helper 2 cells (STAT6 and STAT5A), follicular helper T cells (BCL6), Tregs (FOXP3, STAT5B and TGFB1) and exhausted T cells (PDCD1, CTLA4, LAG3 and HAVCR2). Interestingly, we found that TFAP4 was negatively correlated with the expression levels of PD1 (PDCD1) and CTLA4 in BLCA, COAD, LGG, LUSC, PCPG, SKCM, TGCT, UCEC and UVM, but positively correlated with KIRC and LIHC, suggesting that TFAP4 might regulate the immune response in these cancer types.

### 3.5 Correlation analysis with ESTIMATE score, TMB and MSI

The ESTIMATE method was developed to calculate the immune and stromal scores of cancer tissues. Using the ESTIMATE method, we calculated the immune, stromal and estimate scores, after which we evaluated the relationship between immune/stromal scores and TFAP4 expression. Figure 7D shows the typical results for HCC, in which TFAP4 expression is significantly correlated with both stromal and estimate scores. The detailed correlation results are summarized in Table 2.

**Table 2:** Correlation analysis of TFAP4 expression with ESTIMATE scores

Cancer	*P*‐value		
	StromalScore	ImmuneScore	ESTIMATEScore
ACC	0.935957	0.37771	0.584187
BLCA	0	2.24E‐10	2.11E‐14
BRCA	0	2.03E‐09	0
CESC	8.75E‐05	0.14378	0.004065
CHOL	0.914703	0.637162	0.8321
COAD	9.34E‐09	1.08E‐13	4.50E‐12
DLBC	0.405008	0.109136	0.139427
ESCA	0.004661	0.004048	0.001886
GBM	7.49E‐10	4.33E‐10	8.71E‐11
HNSC	7.24E‐07	0.008614	6.67E‐05
KICH	0.05225	0.54298	0.223598
KIRC	0.189648	0.25622	0.930842
KIRP	6.43E‐07	0.001451	6.05E‐05
LAML	8.16E‐05	5.94E‐06	1.43E‐05
LGG	0	3.52E‐07	1.65E‐13
LIHC	2.31E‐06	0.068378	0.001169
LUAD	8.38E‐16	2.74E‐08	1.04E‐13
LUSC	0	0	0
MESO	0.00277	0.000435	0.000154
OV	5.92E‐09	2.14E‐08	2.01E‐10
PAAD	2.52E‐07	0.009034	6.20E‐05
PCPG	0.933774	0.049116	0.369698
PRAD	0.000594	0.004838	0.000955
READ	0.000191	4.01E‐06	3.88E‐06
SARC	1.15E‐08	0.001775	1.22E‐05
SKCM	2.40E‐10	1.68E‐12	2.90E‐13
STAD	4.06E‐06	0.010348	8.30E‐05
TGCT	0.000637	7.35E‐06	1.36E‐07
THCA	0.001201	0.00509	0.001172
THYM	0.014624	0.129059	0.991124
UCEC	1.50E‐11	8.77E‐10	2.64E‐12
UCS	0.896022	0.156406	0.478438
UVM	0.017035	0.003438	0.003283

**Figure 8:**
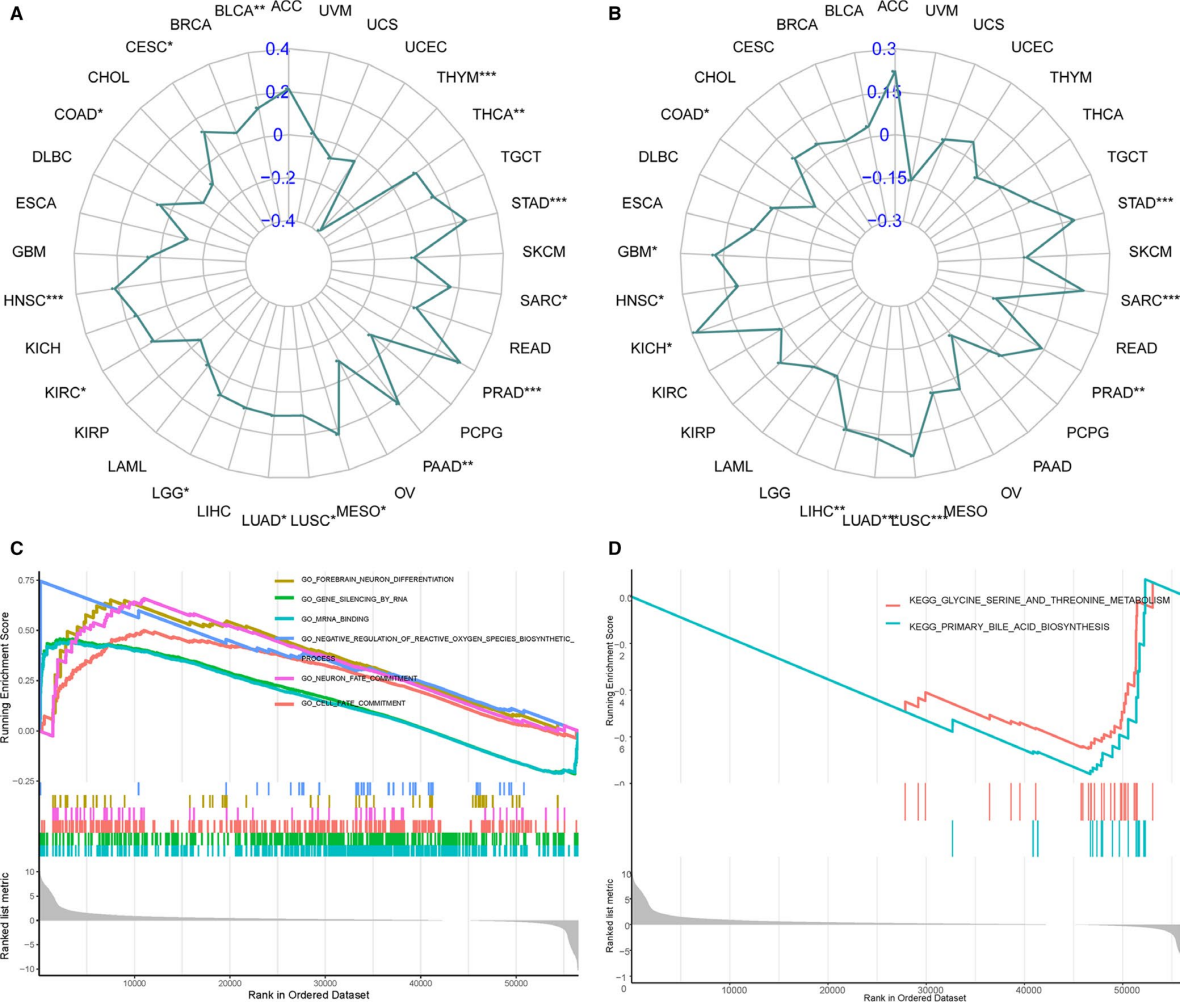
Correlation of TFAP4 expression with TMB and MSI, and the subsequent GSEA analysis. (A) Radar chart displays the overlap between TFAP4 and TMB. Blue number represents the Spearman correlation coefficient. (B) Radar chart displays the overlap between TFAP4 and MSI. Blue number represents the Spearman correlation coefficient. (C) GSEA shows the top GO terms or KEGG pathways correlated with TFAP4 expression in HCC. *P < .05; **P < .01; ***P < .001

Moreover, the association between TMB/MSI and TFAP4 expression was also evaluated, as seen in Table 3. We found that TFAP4 expression was positively correlated with TMB in CESC (*P* = .030), BLCA (*P* = .005), KIRC (*P* = .023), HNSC (*P* < .001), LUAD (*P* = .013), LGG (*P* = .046), MESO (*P* = .043), LUSC (*P* = .015), PRAD (*P* < .001), PAAD (*P* = .004),STAD (*P* < .001), SARC (*P* = .013) and THCA (*P* = .005), but negatively correlated with COAD THYM (*P* < .001) and COAD (*P* = .026), as seen in Figure 8A. We also found that the TFAP4 level was positively correlated with MSI in HNSC (*P* = .020), GBM (*P* = .030), KICH (*P* = .021), LIHC (*P* = .003), PRAD (*P* = .002), LUAD (*P* < .001), SARC (*P* = .001), LUSC (*P* < .001) and STAD (*P* < .001), but negatively correlated with COAD (*P* = .032), as seen in Figure 8B.

**Table 3:** Correlation analysis of TFAP4 expression with TMB and MSI

Cancer	TMB		MSI	
	Correlation	*P*‐value	correlation	*P*‐value
ACC	0.213990477	.058269	0.221205	.050099
BLCA	0.138340844	.005122	0.037982	.444196
BRCA	0.054822285	.087259	0.012035	.707558
CESC	0.12800435	.030451	0.046697	.431466
CHOL	−0.088367935	.608301	0.057915	.736499
COAD	−0.11221118	.025551	−0.10778	.032229
DLBC	0.059981034	.723559	0.018258	.914584
ESCA	−0.116478286	.142433	0.057006	.473985
GBM	0.046330797	.576053	0.178938	.029554
HNSC	0.218042857	1.04E−06	0.104512	.020414
KICH	0.146133987	.245415	0.285998	.020915
KIRC	0.124706288	.023052	0.007689	.888997
KIRP	−0.057764267	.337264	0.079092	.188562
LAML	0.001201751	.992542	0.008382	.948881
LGG	0.089256972	.045843	−0.00852	.849165
LIHC	0.100357725	.057476	0.154218	.003396
LUAD	0.110989813	.012747	0.165302	.000196
LUSC	0.110235151	.014836	0.224462	5.59E−07
MESO	0.22860519	.042722	0.019671	.863384
OV	−0.090001627	.138736	0.042903	.485128
PAAD	0.23122869	.004282	−0.13079	.109451
PCPG	−0.09261469	.220174	0.040581	.591759
PRAD	0.321266464	4.92E‐13	0.141294	.001873
READ	0.02842207	.746313	−0.07814	.373142
SARC	0.161479303	.013193	0.215262	.000919
SKCM	−0.016322013	.725557	0.009055	.845603
STAD	0.249946559	1.20E‐06	0.193492	.000188
TGCT	0.143339703	.085434	0.073046	.382587
THCA	0.127527734	.005047	0.009191	.840494
THYM	−0.38568753	1.75E‐05	−0.03537	.705023
UCEC	−0.032979509	.450814	0.054971	.208579
UCS	−0.072182054	.597035	0.016918	.901509
UVM	0.017096006	.880358	‐0.15461	.170892

Abbreviations: TMB, tumour mutation burden; MSI, microsatellite instability.

### 3.6 Functional analysis

The biological effect of TFAP4 expression was assessed using GSEA. In HCC, TFAP4 showed significant enrichment in the following GO terms: GO_MRNA_BINDING, GO_GENE_SILENCING_BY_RNA, GO_CELL_FATE_COMMITMENT, GO_NEURON_FATE_COMMITMENT, GO_FOREBRAIN_NEURON_DIFFERENTIATION and GO_NEGATIVE_REGULATION_OF_REACTIVE_OXYGEN_SPECIES_BIOSYNTHETIC_PROCESS. The following KEGG terms also showed significant enrichment: KEGG_PRIMARY_BILE_ACID_BIOSYNTHESIS and KEGG_GLYCINE_SERINE_AND_THREONINE_METABOLISM. These can be seen in Figure 8C and 8D, respectively. The pan‐cancer functional GO and KEGG lists of TFAP4 are available in Tables S9 and S10.

## 4 DISCUSSION

The present study aimed to demonstrate a comprehensive workflow for pan‐cancer analysis and to extensively investigate the role of TFAP4 as it relates to various cancers. Based on our results, we found that TFAP4 expression varied among different types of cancer. We also found that most cancer types had a higher number of TFAP4 alternations, and that abnormal TFAP4 expression served as a prognostic factor in some types of cancer, based on both Cox and KM survival analyses. These cancers included KIRC, KIRP, LIHC, READ, THYM and UVM. More importantly, we predicted that TFAP4 overexpression was associated with cancer immunity, and it was, in fact, found to correlate with TMB and MSI. Bioinformatics analyses were carried out to identify the TFAP4 expression‐associated GO terms and KEGG pathways.

It is important to identify the abnormal expression of genes among different tumour types, and it is even more important to identify tumour‐specific targets or features for individualized treatment, allowing us to increase the chance of successfully treating or curing cancer cases.^26^ Pan‐cancer analysis of TFAP4 is valuable for identifying differential expressions and the role of TFAP4 in many cancer types.^27^, ^28^ Using CCLE and TCGA, data on various types of cancers with large sample sizes were obtained, which aided in the discovery of abnormal expressions of TFAP4 among different types of cancers. Using CCLE, a thorough pan‐cancer cellular analysis can be performed to assess the expression of genes, which may shed light on future cellular experiments. On the other hand, TCGA genomic and survival analyses may provide guidance for clinical implications and future studies. For example, in the present study, we found that TFAP4 indicated a worse prognosis for HCC, which is consistent with our previously published study.^16^ Meanwhile, TFAP4 indicated a worse prognosis for KIRC and KIRP, while TFAP4 predicted a better prognosis for READ, THYM and UVM. Nonetheless, the role of TFAP4 in these cancers still needs to be further investigated.

In recent years, immunotherapy has exhibited an increased efficacy in treating tumours. Notably, the present study has demonstrated that the TFAP4 level was related to cancer immunity. Based on the results of this study, the TFAP4 level was related to the degree of immune infiltration in a variety of cancers. We used HCC as an example for illustration purposes. We found that TFAP4 levels were significantly correlated with the degree of infiltration in CD4 + T cells, CD8 + T cells, B cells, neutrophils, macrophages and dendritic cells, based on TIMER analysis. In addition, the TFAP4 level was found to be significantly correlated with the degree of infiltration in Macrophages M0, Macrophages M2, T cells gamma delta, T cells follicular helper and Mast cells resting, based on CIBERSORT analysis. TFAP4 was also found to be correlated with TIL gene markers, as seen in Figure 7C. ESTIMATE was reported as a metric for evaluating cancer patient prognosis.^29^ Recently, numerous studies have used the ESTIMATE method to assess various tumours, and it has been successfully applied to genomic data. For instance, ESTIMATE is used to predict prognoses in glioblastoma and cutaneous melanoma patients.^30^, ^31^ Using the TCGA cohort, the ESTIMATE approach was utilized to generate immune and stromal scores. We found that TFAP4 was negatively correlated with the ESTIMATE scores.

Gene mutations are the primary cause of cancer formation.^32^ Specific gene mutations may predict patient prognosis and treatment response.^33^, ^34^ The adaptive immune system can identify and detect cancers through somatic mutation‐associated non‐self neoantigens. TMB levels affect immunogenic peptide generation, thus affecting the patient response to immune checkpoint inhibitors.^35^, ^36^ As a result, it is highly important to carry out a thorough investigation on the association of TFAP4 expression with TMB levels in cancer patients, based on the TCGA‐derived matched data with high quality. Moreover, TMB and MSI levels indicate that new antibodies are produced. It has been well reported that numerous patients with high microsatellite instability (MSI‐H) have increased TMB levels.^37^ As discovered by Bonneville et al,^38^ cervical squamous cell and adrenocortical carcinomas that had MSI‐H showed abnormally high mutation frequencies. MSI is a vital index for predicting tumorigenesis and development.^25^ MSI testing has been recommended for all CRC subtypes by the NCCN guidelines, as mortality can be reduced by the early detection of MSI.^39^ PD‐1 inhibitors are highly effective for MSI‐H solid tumours,^40^ and as a result, the FDA has approved the use of Keytruda for MSI‐H solid tumours.^41^ Therefore, both TMB and MSI can be used as predictive factors for the potential efficacy of immunotherapy. In the present study, we found that TFAP4 expression was correlated with both TMB and MSI in COAD, LAML, OV, PCPG, READ, SKCM, THYM, UCS and UVM. However, further studies are required to determine whether TFAP4 can serve as a predictor for the efficacy of immunotherapy in these types of cancers. Taken together, the findings of the present study provide clues for the association between TFAP4 and cancer immunity.

Collectively, our comprehensive pan‐cancer analysis has illustrated the characterization of TFAP4 within cancer cell lines and tissues. Moreover, we have found that TFAP4 can serve as a valuable prognostic biomarker for some types of cancer. Based on the results of the present study, the TFAP4 level is related to cancer immunity. Moreover, our new integrative omics‐based workflow may be adopted to generate hypotheses about novel targets for cancers.

## CONFLICT OF INTEREST

The authors declare that the research was conducted in the absence of any commercial or financial relationships that could be construed as a potential conflict of interest.

## AUTHOR CONTRIBUTIONS

Liu, Li and Chen: Conception and design of the study. Li, Liu, Wang, Sun and Chen: Literature search, generation of figures and tables, and manuscript writing. Liu, Sun, Li, Kong and Wang: Data collection and data analysis and manuscript revision. Li and Chen: Study supervision and manuscript writing.

## Supporting information

Figure S1

Table S1

Table S2

Table S3

Table S4

Table S5

Table S6

Table S7

Table S8

Table S9

Table S10

## Data Availability

Publicly available data sets were analysed in this study. These data can be found at https://tcga.xenahubs.net and https://portals.broadinstitute.org/ccle.

## References

[1] Bray F , Ferlay J , Soerjomataram I , Siegel RL , Torre LA , Jemal A . Global cancer statistics 2018: GLOBOCAN estimates of incidence and mortality worldwide for 36 cancers in 185 countries. CA. 2018;68:394‐424. 30207593 10.3322/caac.21492

[2] Cancer Genome Atlas Research N , Weinstein JN , Collisson EA , et al. The cancer genome atlas pan‐cancer analysis project. Nat Genet. 2013;45:1113‐1120. 24071849 10.1038/ng.2764 PMC3919969

[3] Li W , Chen QF , Huang T , Shen L , Huang ZL , Wu P . Profiles of m(6)A RNA methylation regulators for the prognosis of hepatocellular carcinoma. Oncology Letters. 2020;19:3296‐3306. 32256825 10.3892/ol.2020.11435 PMC7074306

[4] Huang ZL , Li W , Chen QF , Wu PH , Shen LJ . Eight key long non‐coding RNAs predict hepatitis virus positive hepatocellular carcinoma as prognostic targets. World J Gastrointestinal Oncol. 2019;11:983‐997. 10.4251/wjgo.v11.i11.983 PMC6883184 31798779

[5] Cancer Cell Line Encyclopedia C, Genomics of Drug Sensitivity in Cancer C . Pharmacogenomic agreement between two cancer cell line data sets. Nature. 2015;528:84‐87. 26570998 10.1038/nature15736 PMC6343827

[6] Ye Y , Hu Q , Chen H , et al. Characterization of hypoxia‐associated molecular features to aid hypoxia‐targeted therapy. Nat Metabolism. 2019;1:431‐444. 10.1038/s42255-019-0045-8 PMC6980239 31984309

[7] Barger CJ , Branick C , Chee L , Karpf AR . Pan‐cancer analyses reveal genomic features of FOXM1 overexpression in cancer. Cancers. 2019;11(2):251. 10.3390/cancers11020251 PMC6406812 30795624

[8] Li Y , Li L , Wang Z , et al. LncMAP: Pan‐cancer atlas of long noncoding RNA‐mediated transcriptional network perturbations. Nucleic Acids Res. 2018;46:1113‐1123. 29325141 10.1093/nar/gkx1311 PMC5815097

[9] Danaher P , Warren S , Lu R , et al. Pan‐cancer adaptive immune resistance as defined by the Tumor Inflammation Signature (TIS): results from The Cancer Genome Atlas (TCGA). J Immun Cancer. 2018;6:63. 10.1186/s40425-018-0367-1 PMC6013904 29929551

[10] Jones S . An overview of the basic helix‐loop‐helix proteins. Genome Biol. 2004;5:226. 15186484 10.1186/gb-2004-5-6-226 PMC463060

[11] Xinghua L , Bo Z , Yan G , et al. The overexpression of AP‐4 as a prognostic indicator for gastric carcinoma. Med Oncol. 2012;29:871‐877. 21336989 10.1007/s12032-011-9845-8

[12] Wei J , Yang P , Zhang T , et al. Overexpression of transcription factor activating enhancer binding protein 4 (TFAP4) predicts poor prognosis for colorectal cancer patients. Exp Therap Med. 2017;14:3057‐3061. 28912857 10.3892/etm.2017.4875 PMC5585722

[13] Chen C , Cai Q , He W , et al. AP4 modulated by the PI3K/AKT pathway promotes prostate cancer proliferation and metastasis of prostate cancer via upregulating L‐plastin. Cell Death Dis. 2017;8:e3060. 28981098 10.1038/cddis.2017.437 PMC5680569

[14] Gong H , Han S , Yao H , Zhao H , Wang Y . AP4 predicts poor prognosis in nonsmall cell lung cancer. Mol Med Rep. 2014;10:336‐340. 24804973 10.3892/mmr.2014.2209

[15] Song J , Xie C , Jiang L , et al. Transcription factor AP‐4 promotes tumorigenic capability and activates the Wnt/beta‐catenin pathway in hepatocellular carcinoma. Theranostics. 2018;8:3571‐3583. 30026867 10.7150/thno.25194 PMC6037031

[16] Huang T , Chen QF , Chang BY , et al. TFAP4 Promotes hepatocellular carcinoma invasion and metastasis via activating the PI3K/AKT signaling pathway. Dis Markers. 2019;2019:7129214. 31281549 10.1155/2019/7129214 PMC6590577

[17] Li T , Fan J , Wang B , et al. TIMER: A Web Server for Comprehensive Analysis of Tumor-Infiltrating Immune Cells. Cancer Res. 2017;77:e108-e110. 29092952 10.1158/0008-5472.CAN-17-0307 PMC6042652

[18] Li B , Severson E , Pignon JC , et al. Comprehensive analyses of tumor immunity: implications for cancer immunotherapy. Genome Biol. 2016;17:174. 27549193 10.1186/s13059-016-1028-7 PMC4993001

[19] Chen QF , Li W , Wu PH , Shen LJ , Huang ZL . Significance of tumor‐infiltrating immunocytes for predicting prognosis of hepatitis B virus‐related hepatocellular carcinoma. World J Gastroenterol. 2019;25:5266‐5282. 31558872 10.3748/wjg.v25.i35.5266 PMC6761238

[20] Siemers NO , Holloway JL , Chang H , et al. Genome‐wide association analysis identifies genetic correlates of immune infiltrates in solid tumors. PLoS One. 2017;12:e0179726. 28749946 10.1371/journal.pone.0179726 PMC5531551

[21] Danaher P , Warren S , Dennis L , et al. Gene expression markers of Tumor Infiltrating Leukocytes. J Immun Cancer. 2017;5:18. 10.1186/s40425-017-0215-8 PMC5319024 28239471

[22] Pan JH , Zhou H , Cooper L , et al. LAYN Is a prognostic biomarker and correlated with immune infiltrates in gastric and colon cancers. Front Immunol. 2019;10:6. 30761122 10.3389/fimmu.2019.00006 PMC6362421

[23] Yoshihara K , Shahmoradgoli M , Martinez E , et al. Inferring tumour purity and stromal and immune cell admixture from expression data. Nat Commun. 2013;4:2612. 24113773 10.1038/ncomms3612 PMC3826632

[24] Krieger T , Pearson I , Bell J , Doherty J , Robbins P . Targeted literature review on use of tumor mutational burden status and programmed cell death ligand 1 expression to predict outcomes of checkpoint inhibitor treatment. Diag Pathol. 2020;15:6. 10.1186/s13000-020-0927-9 PMC6990470 32000815

[25] Li K , Luo H , Huang L , Luo H , Zhu X . Microsatellite instability: a review of what the oncologist should know. Cancer Cell Int. 2020;20:16. 31956294 10.1186/s12935-019-1091-8 PMC6958913

[26] Andre F , Mardis E , Salm M , Soria JC , Siu LL , Swanton C . Prioritizing targets for precision cancer medicine. Ann Oncol. 2014;25:2295‐2303. 25344359 10.1093/annonc/mdu478

[27] Cao Z , Zhang S . An integrative and comparative study of pan‐cancer transcriptomes reveals distinct cancer common and specific signatures. Sci Rep. 2016;6:33398. 27633916 10.1038/srep33398 PMC5025752

[28] Cava C , Bertoli G , Colaprico A , Olsen C , Bontempi G , Castiglioni I . Integration of multiple networks and pathways identifies cancer driver genes in pan‐cancer analysis. BMC Genom. 2018;19:25. 10.1186/s12864-017-4423-x PMC5756345 29304754

[29] Liu W , Ye H , Liu YF , et al. Transcriptome‐derived stromal and immune scores infer clinical outcomes of patients with cancer. Oncol Lett. 2018;15:4351‐4357. 29541203 10.3892/ol.2018.7855 PMC5835954

[30] Jia D , Li S , Li D , Xue H , Yang D , Liu Y . Mining TCGA database for genes of prognostic value in glioblastoma microenvironment. Aging. 2018;10:592‐605. 29676997 10.18632/aging.101415 PMC5940130

[31] Yang S , Liu T , Nan H , et al. Comprehensive analysis of prognostic immune‐related genes in the tumor microenvironment of cutaneous melanoma. J Cell Physiol. 2020;235:1025‐1035. 31240705 10.1002/jcp.29018

[32] Martincorena I , Campbell PJ . Somatic mutation in cancer and normal cells. Science. 2015;349:1483‐1489. 26404825 10.1126/science.aab4082

[33] Sanz‐Garcia E , Argiles G , Elez E , Tabernero J . BRAF mutant colorectal cancer: prognosis, treatment, and new perspectives. Ann Oncol. 2017;28:2648‐2657. 29045527 10.1093/annonc/mdx401

[34] Allegra CJ , Rumble RB , Hamilton SR , et al. Extended RAS gene mutation testing in metastatic colorectal carcinoma to predict response to anti‐epidermal growth factor receptor monoclonal antibody therapy: American society of clinical oncology provisional clinical opinion update 2015. J Clin Oncol. 2016;34:179‐185. 26438111 10.1200/JCO.2015.63.9674

[35] Wu HX , Wang ZX , Zhao Q , et al. Tumor mutational and indel burden: a systematic pan‐cancer evaluation as prognostic biomarkers. Ann Trans Med. 2019;7:640. 10.21037/atm.2019.10.116 PMC6944566 31930041

[36] Havel JJ , Chowell D , Chan TA . The evolving landscape of biomarkers for checkpoint inhibitor immunotherapy. Nat Rev Cancer. 2019;19:133‐150. 30755690 10.1038/s41568-019-0116-x PMC6705396

[37] Chalmers ZR , Connelly CF , Fabrizio D , et al. Analysis of 100,000 human cancer genomes reveals the landscape of tumor mutational burden. Genome Med. 2017;9:34. 28420421 10.1186/s13073-017-0424-2 PMC5395719

[38] Bonneville R , Krook MA , Kautto EA , et al. Landscape of microsatellite instability across 39 cancer types. JCO Precision Oncol. 2017;1:1‐15. 10.1200/PO.17.00073 PMC5972025 29850653

[39] Benson AB 3rd , Venook AP , Cederquist L , et al. Colon cancer, version 1.2017, NCCN Clinical Practice Guidelines In Oncology. J Natl Comp Cancer Network. 2017; 15: 370‐398. 10.6004/jnccn.2017.0036 28275037

[40] Diaz LA Jr , Le DT . PD‐1 blockade in tumors with mismatch‐repair deficiency. New Engl J Med. 2015;373:1979. 10.1056/NEJMc1510353 26559582

[41] Yu Y . Molecular classification and precision therapy of cancer: immune checkpoint inhibitors. Front Med. 2018;12:229‐235. 29209918 10.1007/s11684-017-0581-0

