# Supplementary figures and images for: An integrated pan‐cancer analysis of TFAP4 aberrations and the potential clinical implications for cancer immunity
Source: J Cell Mol Med. 2020 Dec 29;25(4):2082–97. doi: 10.1111/jcmm.16147 (PMC7882993; doi:10.1111/jcmm.16147)

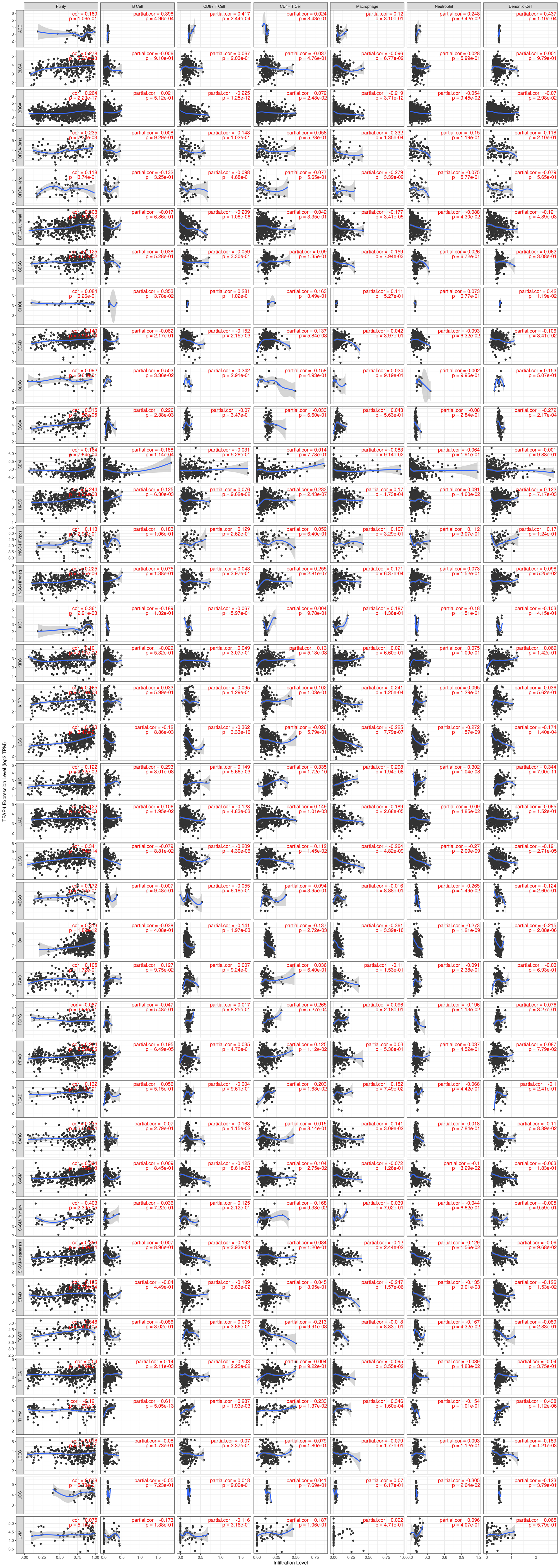

Supplement: Supplementary file 1 — Figure S1 [file JCMM-25-2082-s001.tif]
